# Knowing for whom the bell tolls: acting locally and thinking globally. Brazil, Latin America and the Global Burden of Diseases, 2015: Sabendo por quem os sinos dobram: atuando localmente e pensando globalmente. Brasil, América Latina e a carga global de doenças, 2015

**DOI:** 10.1590/1516-3180.2016.1346171016

**Published:** 2016-06-03

**Authors:** Paulo Andrade Lotufo

**Affiliations:** I MD, DrPH. Full Professor, Department of Internal Medicine, Faculdade de Medicina da Universidade de São Paulo (FMUSP), São Paulo (SP), Brazil.

*"No man is an Iland, intire of it selfe; every man is a peece of the Continent, a part of the maine; if a Clod bee washed away by the Sea, Europe is the lesse, as well as if a Promontorie were, as well as if a Mannor of thy friends or of thine owne were; any mans death diminishes me, because I am involved in Mankinde; And therefore never send to know for whom the bell tolls; It tolls for thee" - John Donne (1572-1623) Devotions upon Emergent Occasions*

The Global Burden of Diseases, Injuries and Risk Factors (GBD) study is an initiative from the Institute for Health Metrics and Evaluation of the University of Washington, with unrestricted grants from the Melinda and Bill Gates Foundation. The GBD study has been developing two concepts to enable geographical and temporal trend comparisons. These are the number of years of life lost (YLLs) and number of years of life lived with disability (YLDs). Combination of these two indicators reveals the number of disability-adjusted life-years (DALYs). Moreover, the GBD study is publishing data relating to risk factors. A more detailed description of these indexes can be found in the special issue of The Lancet dated October 7, 2016. The data presented below are based on the results of the articles addressing YLL,^1^ YLD,^2^ DALY^3^ and risk factors.^4^

The world can be divided geopolitically in several ways, but there is one grouping that is applied by most agencies: the category of Latin American and Caribbean countries. It represents 50 independent countries and a few colonies with 640 million inhabitants. However, two-thirds of this population and three-quarters of the gross product is concentrated in four countries: Brazil, Mexico, Colombia and Argentina. We will discuss the differences in the GBD indexes among these four countries (Table 1).

**Table 1:**
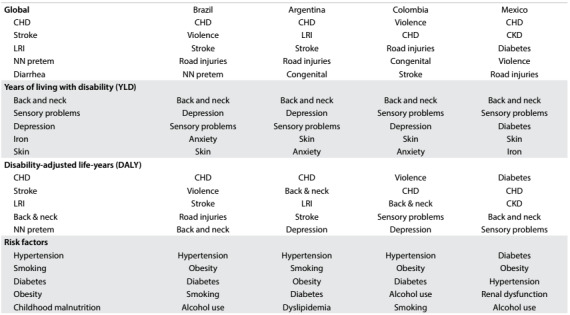
Description of the causes of years of life lost (YLL), years of living with disability (YLD) and disability-adjusted life-years (DALY) for all countries and for Brazil, Argentina, Colombia and Mexico

CHD = coronary heart disease; LRI = lower respiratory infection; CKD = chronic kidney disease; NN pretem = neonatal preterm disorders sensory disabilities.

## MORTALITY

The ranking of the five most important causes of YLLs shows how important the burden of coronary heart disease and stroke is, both worldwide and in these four countries, for both sexes. The coronary heart disease (CHD) values were similar among the four countries, but the risk of death due to stroke was significantly higher in Brazil for men and women than in Argentina, Colombia and Mexico. 

Two particular causes in Latin American and the Caribbean countries are violence and road traffic, which are ranked within the top five causes of deaths, in contrast to the global data. The road traffic death rate was highest in Brazil (24 per 100,000) followed by Colombia (17), Mexico (17) and Argentina (14). However, the proportion of pedestrian deaths was greater in Colombia (62%) followed by Mexico (44%), Brazil (35%) and Argentina (28%). Violence is a characteristic of Latin American and Caribbean countries: the age-adjusted homicide rates (per 100,000) in 2015 were 34 in Colombia, 28 in Brazil and 17 in Mexico, but only 6 in Argentina. Another four Latin American and Caribbean countries had higher rates that these: Venezuela, El Salvador, Honduras and Guatemala. The proportion of homicides due to firearms was significantly different: Colombia (78%), Brazil (69%), Mexico (59%) and Argentina (50%).

Another difference observed was the importance of chronic kidney disease (CKD) and diabetes in Mexico. The risk of death due to CKD in Mexico was three times higher than in Argentina, Brazil and Colombia.

## YEARS LIVING WITH DISABILITY

Both globally and in these four Latin American and Caribbean countries, an impressive amount of time is lost through disability relating to back and neck disorders, sensory problems, anxiety and depression and skin complaints. However, in contrast to the other countries, diabetes appears as one of the greatest causes of YLDs in Mexico.

## DALYs

DALYs are derived from a combination of YLLs and YLDs. They show that CHD is the most important cause in Brazil and Argentina, as also seen worldwide, and that it is the second biggest cause in Colombia and Mexico. Violence is the top cause in Colombia and diabetes leads the causes of DALYs in Mexico. Neck and back pain and psychiatric disorders have similar impact with regard to DALYs, compared with cardiovascular diseases.

## RISK FACTORS

Hypertension is the most important risk factor globally, and in Brazil, Argentina and Colombia, but not in Mexico. The combination of obesity and diabetes is important in all Latin American and Caribbean countries, especially in Mexico, with an association with chronic kidney disease. Smoking was ranked second in the world and in Argentina, fourth in Brazil and fifth in Colombia. Alcohol use was classified as one of the five most important risk factors in Brazil, Colombia and Mexico.

## WHAT IS NEW IN GBD 2015?

A relatively large amount of information about mortality and DALYs is available, but the impact of non-lethal conditions on increasing YLDs and DALYs deserves more attention from the Brazilian health authorities. São Paulo Medical Journal dedicated three editorials^5^^,^^6^^,^^7^ and four articles^8^^,^^9^^,^^10^^,^^11^ to the important topic of lumbar pain. Depression, anxiety and skin disorders need to be more appropriately ranked as priorities for research worldwide, and the focus of medical care needs to be shifted to primary care, such that it is not managed only by medical specialists.

## WHAT ARE THE PRIORITIES FOR ARGENTINA, BRAZIL, COLOMBIA AND MEXICO?

In relation to the epidemiological profile of chronic diseases, GBD 2015 makes it possible to establish five priorities in Latin America and Caribbean countries:

1. Alcohol intake reduction: High alcohol consumption is strongly associated with car and motorcycle crashes. Latin America and Caribbean governments are notoriously lenient with regard to punishing individuals who drive under the influence of alcohol beverages. Moreover, deadly interpersonal violence is also related to alcohol abuse.^12^^,^^13^
2. Hypertension: Reduction of high blood pressure can reduce racial and social disparities regarding deaths due to cardiovascular diseases. In all countries, it is essential:
1. to improve awareness, treatment and control of hypertension;
2. to reduce sodium intake;
3. to spread the use of automatic sphygmomanometer devices with greater precision and accuracy;^14^^,^^15^
4. to deliver antihypertensive drugs free of charge (shifting from thiazides to chlorthalidone or indapamide); and
5. to take special care of individuals with resistant hypertension, who account for 3% of the adult population.^16^
3. CHD secondary prevention: CHD is the leading cause of death, and the first cause of DALYs in Argentina and Brazil and the second one in Colombia and Mexico. The high prevalence rates of people with CHD implies to amplify national program addressing secondary prevention including smoking quitting and free delivery of aspirin, statins, and angiotensin-converting-enzyme (ACE) inhibitors.^17^
4. Obesity-diabetes prevention: The prevalence of overweight and obesity in Mexico has been increasing at an alarming rate, with high rates of adverse outcomes relating to diabetes. However, although it is easy to put forward proposals for curbing the obesity epidemic (reduction of calorie intake and increase of physical activity), such proposals are very ineffective. One strategy should be to focus on childhood, so as to avoid obesity among the next generation of teenagers and young adults.^18^
5. CKD screening: Renal failure in Mexico and Central America leads to high rates of DALYs. This is thought to be due to the high prevalence rate of diabetes in Mexico and of Mesoamerican nephropathy due to heat stress or intoxication with herbicides.^19^^,^^20^ Although there is no consensus regarding CKD screening through determination of serum creatinine and urinary albumin, verification of the cost-effectiveness of early diagnosing of CKD is urged.^21^^,^^22^

## CONCLUSION

The complexity of the "health-disease" process implies that there is a need to plan and think globally, while acting locally. The most important lesson is that there is only one island: the Earth. And also, the bell tolls for everyone.
